# Management of neurofibromatosis type 2 and schwannomatosis associated peripheral and intraspinal schwannomas: influence of surgery, genetics, and localization

**DOI:** 10.1007/s11060-022-04061-0

**Published:** 2022-06-30

**Authors:** Isabel Gugel, Florian Grimm, Marcos Tatagiba, Martin U. Schuhmann, Julian Zipfel

**Affiliations:** 1 Department of Neurosurgery, University Hospital Tübingen, 72076 Tübingen, BW Germany; 2 Centre of Neurofibromatosis and Rare Diseases, University Hospital Tübingen, 72076 Tübingen, BW Germany; 3 Section of Pediatric Neurosurgery, University Hospital Tübingen, 72076 Tübingen, BW Germany

**Keywords:** Schwannoma, Neurofibromatosis type 2, Schwannomatosis, Surgery, Peripheral nerve sheath tumor, Spinal schwannoma

## Abstract

**Introduction:**

Peripheral and intraspinal schwannomas are common and clinically complex pathologies in patients with Neurofibromatosis Type 2 (NF2) and Schwannomatosis (SWNT). Functional preservation and pain relief are the major goals in treating these tumors.

**Methods:**

This retrospective observational study investigates the clinical and functional outcome of 205 operated peripheral (n = 148, 72%) and intraspinal (n = 57, 28%) schwannomas in 85 patients (53 NF2, 32 SWNT) treated at our department between 2006 and 2017. Associated factors such as genetics, age, and location were evaluated.

**Results:**

Persisting drug-resistant pain was the most common symptom (84%, n = 173) and indication for surgery (54%, n = 110). Improvement in pain intensity was postoperatively seen in 81%. Peripheral nerve schwannomas exhibited worse pain intensity preoperatively compared to intraspinal lesions (*p* = 0.017 NF2, *p* = 0.029 SWNT). More total resections could be achieved in 93% of SWNT vs. 82% of NF2-associated tumors, *p* = 0.030). NF2 patients with intraspinal lesions were more neurologically affected (*p* < 0.05). Perioperative comparison of both tumor syndromes showed more neurological deficits (*p* = 0.027), and less pain (*p* = 0.024) in NF2-associated tumors. Mosaic NF2 patients had worse pain levels before surgery, and SWNT patients had a worse neurological function and more pain compared to non-mosaic or non-mutated cases.

**Conclusions:**

Resection of peripheral and intraspinal schwannomas is an effective and low-risk treatment in both NF2 and SWNT. Patients with severe pain have a particular benefit from surgical treatment. Intraspinal lesions are associated with worse neurological function whereas peripheral lesions showed a higher pain intensity. The influence of mutations needs to be further investigated in larger cohorts.

**Supplementary Information:**

The online version contains supplementary material available at 10.1007/s11060-022-04061-0.

## Introduction

Peripheral nerve schwannomas (PNS) can either occur as solitary lesions or are less often associated with a tumor predisposition syndrome such as schwannomatosis (SWNT) or neurofibromatosis type 2 (NF2). Both syndromes are inherited in an autosomal-dominant manner and are caused by the inactivation of tumor suppressor genes *such as the LZTR1* and *SMARCB1* gene in SWNT and the *NF2 gene* in the case of NF2, all of them located on chromosome 22. Genotype–phenotype correlations are known mainly for NF2 [1] and rarely for SWNT [2].

Schwannomatosis is characterized by the presence of multiple (non-intradermal) PNS, the exclusion of bilateral vestibular schwannomas (VS), and can rarely (5%) be associated with meningiomas [3, 4] or unilateral vestibular schwannomas [5]. Patients with SWNT most frequently  present between the second and  fourth decade of life and affected patients preferentially exhibit localized or diffuse pain [4, 6] as well as an asymptomatic mass rather than focal neurological deficits [7]. SWNT-associated schwannomas affect usually either peripheral nerves of the whole body, occur segmentally limited to one extremity, or strictly unilaterally (one body half) [3].

Typical and pathognomonic for NF2′ are bilateral VS and other peripheral or central nervous system lesions such as meningiomas, spinal ependymomas, non-VS schwannomas, and ocular (e.g. cataracts) or cutaneous findings. Cutaneous schwannomas or subcutaneous (palpable or symptomatic) PNS are—apart from ocular findings—the most common presenting symptom/pathology in young NF2 patients [8, 9]. PNS may also be a potentially associated factor for tumor growth rate in NF2-associated VS [10].

Due to their multiplicity or accompanying comorbidities in these syndromes, the management of phacomatosis-associated PNS is complex. Apart from conservative treatment strategies including pain management, surgery is still the most common and curative therapy. Indications for surgery for phacomatosis-related PNS are similar to sporadic forms and include significant neurological impairment, persisting, distressing and drug-resisting pain, disability, and disfigurement due to tumor masses, as well as significant tumor growth with/without suspicion for malignancy. Radiation therapy has been insufficiently studied regarding its effect on e.g. pain, but is hardly ever used because of the low but present risk of possible malignant transformation, especially in the association of a tumor predisposition syndrome. The major surgical goal is functional preservation with the aim of total tumor removal under local or continuous neurophysiological guidance and ultrasound control.

The present study aimed to investigate the surgical outcome (pain intensity, motor, and sensory function) in SWNT- and NF2-associated PNS under consideration of possible associated factors (genetics, localization, age at time of surgery).

In the following work, intraspinal schwannomas were considered as a subtype of PNS.

## Materials and methods

### Patients and clinics

A total of 85 patients with either NF2 (53 patients) or SWNT (32 patients) and 205 histologically confirmed peripheral (n = 148) and intraspinal (n = 57) nerve schwannomas were included in this retrospective analysis. Patients who were operated on and followed up between 2006 and 2017, at the Department of Neurosurgery and Centre of Neurofibromatosis in Tübingen were included (ICD codes D36.1 or Q85.0). Diagnosis of NF2 and SWNT was established using the published consensus criteria for both diseases [11, 12].

Microsurgical removal was performed in form of intracapsular tumor resection to preserve sensory and motor nerve fascicles. We distinguished between total and partial resection extents. The former involves a complete removal of the tumor and the affected fascicle with preserving the tumor capsule while partial resection describes varying percentages resection extents with a remaining residual tumor.

Direct nerve stimulation (for peripheral nerve lesions) of motor fibers or continuous (for intraspinal lesions) neurophysiological monitoring (SEP, MEP, EMG) was used. The indications for surgery were (1) focal neurological deficits and/or (2) significant tumor growth progression (3) and/or persisting and drug-resistant pain.

No tumor was previously irradiated or has received preoperative biopsy. All tumors were histologically confirmed as schwannoma WHO grade I.

Mutation analysis from blood (11 NF2 and 7 SWNT) and tumor (7 NF2 and 16 SWNT) was performed in 41 (in 18 NF2 and 23 SWNT) patients. The remaining 44 patients or their legal guardians refused it. In the absence of a mutation in the blood DNA, genetic analysis was completed on two independently located tumors within an individual for the presence of a mosaic. Thus, 14 NF2-associated and 32 SWNT-associated tumors were examined for this purpose.

Data of 12 tumors of 10 pediatric patients (8 patients with NF2 and two with schwannomatosis) were part of a prior (published) study and were included in this cohort [13].

Patients’ pre-and postoperative clinical evaluation included a full medical history, general physical and symptom-based neurologic examination, magnetic resonance imaging, as well as peripheral nerve ultrasound. The latter has been used preoperatively in peripheral lesions to localized non-palpable or visible tumors and to verify the exact nerve relationship. In the case of intraspinal schwannomas, it was intraoperatively utilized to verify the tumor extension within the borders of the approach.

Electrophysiology was used as a routine diagnostic to evaluate the surgical outcome and to distinguish the symptomatology from other possible causes to better differentiate between intraspinal from peripheral schwannomas. Furthermore, the extent of resection was controlled by intraoperative monitoring, particularly for intraspinal lesions. Direct nerve stimulation helped to identify functional nerve fibers.

To classify and rate motor and sensory function as well as pain intensity we used the Medical Research Council Scale for Muscle Strength (MCR) [14], the sensory rating scale (SRS) [15], and a four-point Verbal Rating Scale (VRS) with the words “no pain”, “slight pain”, “moderate pain”, and “severe pain” for measuring pain intensity [16] as illustrated and explained in the Supplementary Tables 5 and 6. Pain impression was evaluated just for the surgical outcome, not for the clinical or differential characterization, and was attributable to the palpable, imaging, or sonographic schwannoma finding. It occurred either in acute or chronic progressive time and was dermatome-related in form of radiating pain. Postoperative evaluation was performed within the first postoperative control after 3 months.

Data for evaluation of function and pain was missing or incomplete in 4 (total number 133) NF2—and one (total number 72) SWNT –associated tumor(s) (NF2: lack of scoring values of pre- and postoperative MRC in 3 and postoperative SRS and VRS in one tumor; SWNT: lack of scoring values of pre-and postoperative SRS and postoperative VRS in one tumor).

### Data evaluation

Statistical analysis was performed in SPSS (IBM SPSS Statistics for Windows, Version 22.0., IBM Corp, Armonk, NY, USA).

A Mann–Whitney U test was run for each NF2 and SWNT associated schwannomas to determine if there were differences in the independent variables (motor and sensory function and pain) between tumors with mutations (“NF2 mosaic” vs. “NF2 non-mosaic” mutations, “SWNT mutations all” vs. “SWNT non-mutations” and “intraspinal” vs. “peripheral lesions” in both disease types). For the comparison of tumors with NF2 mutations, all mosaic tumors were compared to all tumors exhibiting NF2 gene mutations (independent of the mutation subtype) in the blood DNA (e. g. nonsense, frameshifting, splicing, large genome alterations, and deletions). For SWNT tumors, the comparison between all detectable SWNT-related mutations (e. g. LZTR1, SMARCB1, ERBB2) in both blood and/or tumor DNA compared to tumors without mutation detection was established.

A Kruskal–Wallis H test was run to determine if there were differences in motor, sensory and pain rating scale between the five localization categories 1–5 (see Table 1) in NF2- and SWNT-related schwannomas. Distributions of rating scales were not similar for all groups, as assessed by visual inspection of a boxplot.

**Table 1:** Demographic data of 85 operated peripheral and intraspinal schwannoma patients (205 tumors)

	NF2	SWNT
No. of patients/tumors	53/133	32/72
Sex (no of female/male)	27/26	17/15
Total follow-up in months (mean ± SD, range)	43 ± 34, 0–121	51 ± 43, 0–135
Age at time of surgery in years (mean ± SD, range)	25 ± 11, 12–62, n = 133	46 ± 14, 16–81, n = 72
Location categories of peripheral (1–4) and intraspinal (5) schwannomas (No)		
Category 1: Head/face/neck/brachial plexus	36 ≙ 27%	16 ≙ 22%
Category 2: Upper extremity	22 ≙ 17%	12 ≙ 17%
Category 3: Lower extremity	24 ≙ 18%	15 ≙ 21%
Category 4: Torso (thorax/abdomen/pelvic/back)	14 ≙ 11%	9 ≙ 13%
Category 5: intraspinal	37 ≙ 28%	20 ≙ 28%
Family history of NF2/Schwannomatosis (yes/no)	3/50	1/31
Detected NF2/SWNT-related gene mutation types (no)	In 10 patients	In 7 patients
*NF2* Splicing mutations	1	–
*NF2* Nonsense mutations	3	–
*NF2* Frameshift mutations	4	–
*NF2* Large deletion	2	–
*SMARCB1*	7	2
*LZTR1*	–	4
*ERBB2*	–	1
Mosaic		
* NF2*	7	–
*LZTR1*	–	1
No mutation was detected in blood and tumor DNA	1	15
NA	35	8
Indication for surgery (no tumors)		
Pain	64 ≙ 48%	45 ≙ 63%
Tumor growth progression	30 ≙ 23%	15 ≙ 21%
Focal neurological (sensory and/or motor) deficits	39 ≙ 29%	11 ≙ 15%
Suspicion of malignancy	–	1 ≙ 1%
Resection amount (no of tumors)		
Total	109 ≙ 82%	67 ≙ 93%
Partial	24 ≙ 18%	5 ≙ 7%

*No* number, *SD* standard deviation, *NF2* neurofibromatosis Type 2, SWNT schwannomatosis, *SMARCB1 SWI/SNF*-related matrix-associated actin-dependent regulator of chromatin subfamily B member 1, *LZTR1* leucine-zipper-like transcriptional regulator 1, *ERBB2* erb-b2 receptor tyrosine kinase 2

A Mann–Whitney U and Wilcoxon signed-ranked test was run to determine if there were differences in the independent variables (pre-and postoperative MCR, SRS, VRS, and Age at time of surgery in years) between patients/tumors with relation to NF2 (“NF2 associated”) or with SWNT (“SWNT associated”).

## Results

### Patients, tumors, and clinics

Detailed demographic and clinical data are summarized in Table 1.

Of all operated tumors, 148 (72%) were peripheral (each 72% NF2 ≙ 96 and SWNT ≙ 52 cases) and 57 (each 28%, ≙ 37 NF2 and ≙ 20 SWNT cases) of intraspinal location. In both NF2 and SWNT, peripheral nerve schwannomas were most often located in the facial/cervical area (n = 52 ≙ 35%) followed in descending order in the lower extremity (n = 39 ≙ 26%), upper extremity (n = 34 ≙ 23%), and lastly the torso (n = 23 ≙ 16%).

7/18 NF2 and 1/23 SWNT patients were identified to be mosaic cases with positive tumor DNA analysis and in 1/18 NF2 and 15/23 SWNT patients no mutation could be detected neither in blood nor in tumor DNA.

In the majority of tumors (n = 174 ≙ 85%), total resection was achieved, whereas only in 29 tumors (≙ 15%) partial resection could be accomplished due to the risk of a persisting functional loss, e. g. because of fragile or impaired feedback of electrophysiological monitoring.

Most of the patients suffered from persisting and medication resistant pain in both entities (total: n = 173, 84%; NF2: n = 105/133 ≙ 79%; SWNT: n = 68/72 ≙ 94%) followed by sensory (total: n = 80, 39%; NF2: n = 56/133 ≙ 42%; SWNT: n = 24/72 ≙ 33%) and motor (total: n = 68, 34%; NF2: 48/130 ≙ 37%; SWNT: n = 20/72 ≙ 28%) deficits before surgery.

Therefore, pain was the most common indication for surgery for both NF2 (48%) and SWNT (63%) associated schwannomas. This was followed by focal neurological deficits (29%) and growth progression (23%) in NF2-associated schwannomas and by growth progression (21%) and focal neurological deficits (15%) in SWNT-associated schwannomas. Only one SWNT-related schwannoma was operated on due to a radiological suspicion of a beginning malignant transformation, which could histologically not be confirmed.

Motor function could be improved in 15% (n = 20)/8% (n = 6) maintained in 81% (n = 105)/83% (n = 59) and worsened in 3% (n = 4)/8% (n = 6) after surgery in NF2-/SWNT-associated tumors. Postoperative sensory function improved in 11% (n = 14)/10% (n = 7), remained stable in 86% (n = 114)/87% (n = 62) and decreased in 16% (n = 5)/3% (n = 2) of NF2-/SWNT-related cases. Lastly, pain intensity improved in 75% (n = 99)/92% (n = 65), was maintained in 24% (n = 32)/7% (n = 5), and worsened in 1% (n = 1)/1% (n = 1) of NF2-/SWNT-associated tumors.

Overall, the complication rate was low, 202 cases had uneventful peri- and postoperative course (99%), and only in 3 cases (1%) complications were observed (two minor postoperative hematomas and one wound infection).

### Association of mutations and location of NF2-related schwannomas and parameters

Distributions of the motor (MRC), sensory (SRS), and pain (VRS) rating scores were similar in the groups “NF2 mosaic” and “NF2 non-mosaic” and in the groups “NF2 associated spinal” and “NF2 peripheral”, as assessed by visual inspection. Median scores for MRC, SRS, and VRS were statistically significantly (*p* < 0.05) different in all comparison groups.

Detailed values are outlined in Table 2. Mosaic NF2 cases seem to have a better preoperative but worse postoperative motor and sensible function and more pain before and after surgery than tumors in non-mosaic NF2 patients. But only the higher preoperative VRS rank scores in mosaic NF2 patients were statistically significant (*p* = 0.017).

**Table 2:** The difference in rating parameters between tumors in mosaic vs. non-mosaic NF2 patients and intraspinal vs. peripheral NF2-related schwannomas

Variable	*U*	z	*p*	Mean rank/median NF2 mosaic	Mean rank/median NF2 non-mosaic
Preoperative MRC	115	0.402	0.837	15.88/5, n = 13	15.21/5, n = 17
Postoperative MRC	109	− 0.145	0.967	15.38/5, n = 13	15.59/5, n = 17
Preoperative SRS	117	0.390	0.805	16/5, n = 13	15.12/5, n = 17
Postoperative SRS	92	− 1.138	0.432	14.04/5, n = 13	16.62/5, n = 17
Preoperative VRS	167	2.483	**0.017**	19.81/3, n = 13	12.21/2, n = 17
Postoperative VRS	156	2.558	0.059	18.96/0, n = 13	12.85/0, n = 17

Variable	*U*	z	*p*	Mean rank/median NF2 intraspinal	Mean rank/median NF2 peripheral
Preoperative MRC	975	− 4.935	** < 0.001**	44.77/4, n = 41	75.05/5, n = 89
Postoperative MRC	1366	− 2.557	**0.011**	54.66/4.5, n = 40	69.65/5, n = 89
Preoperative SRS	1535	− 2.150	**0.032**	57.7/4, n = 43	71.44/5, n = 90
Postoperative SRS	1732	− 0.862	0.389	62.74/4.5, n = 42	68.26/5, n = 90
Preoperative VRS	1597	− 1.750	0.080	59.15/2, n = 43	70.75/3, n = 90
Postoperative VRS	2293	2.143	**0.032**	76.10/1, n = 42	62.02/0, n = 90

*p* < 0.05*.* Significant *p* values between the two groups are written in bold. The higher the mean rank values for motor and sensory classification and the lower the mean rank values for pain classification the better the neurological status and the less pain. This is also valid for the following tables

*MRC* medical research council scale for muscle strength [14], *SRS* sensory rating scale [14], *VRS* verbal rating scale for measuring pain intensity [16]

Peripheral schwannomas showed significant better pre- and postoperative motor (*p* < 0.001*, p* = 0.011), and preoperative sensory function (*p* = 0.032) and less pain after surgery (*p* = 0.032) compared to intraspinal NF2-associated tumors. A tendency towards better postoperative sensory function but higher preoperative pain intensity scores were seen in peripheral NF2-associated tumors (*p* > 0.05).

### Association of mutations and location of SWNT-related schwannomas and parameters

Distributions of the motor (MRC), sensory (SRS), and pain (VRS) rating scores were similar in the groups “SWNT mutations” and “SWNT non-mutations” and in the groups “SWNT associated spinal” and “SWNT peripheral”, as assessed by visual inspection. Median scores for MRC, SRS, and VRS were statistically significantly (*p* < 0.05) different in all comparison groups. Schwannomas of SWNT patients carrying mutations showed more impaired pre-and postoperative MCR and SRS scales (category ≤ 4) and fewer cases with better pain scores before and after surgery compared to those cases without mutations.

Detailed values are outlined in Table 3. In summary, tumors exhibiting no mutation showed significant better pre- and postoperative motor (*p* = 0.019*, p* = 0.004) and sensory function (*p* = 0.001*, p* = 0.003) and less pain intensity (*p* = 0.007*, p* = 0.001). Peripheral SWNT-associated tumors tended to display better motor but worse sensory pre- and postoperative function as well as less pain intensity after surgery (*p* > 0.05) compared to intraspinal lesions. Significant worse preoperative pain intensity scores were seen in peripheral SWNT-associated tumors (*p* = 0.029).

**Table 3:** The difference in rating parameters between tumors with vs. without SWNT-related mutations and intraspinal vs. peripheral SWNT-related schwannomas

Variable	*U*	z	*p*	Mean rank/median SWNT mutations	Mean rank/median SWNT non-mutations
Preoperative MRC	311	− 2.348	**0.019**	25.52/5, n = 23	34.32/5, n = 38
Postoperative MRC	275	− 2.905	**0.004**	23.96/5, n = 23	35.26/5, n = 38
Preoperative SRS	247	− 3.203	**0.001**	22.73/5, n = 22	35.0/5, n = 38
Postoperative SRS	267	− 2.942	**0.003**	23.61/5, n = 22	34.49/5, n = 38
Preoperative VRS	596	2.696	**0.007**	37.91/3, n = 23	26.82/2, n = 38
Postoperative VRS	603	3.203	**0.001**	38.89/0, n = 22	25.64/0, n = 38

Variable	*U*	Z	*p*	Mean rank/median SWNT intraspinal	Mean rank/median SWNT peripheral
Preoperative MRC	451	− 1.097	0.273	33.08/5, n = 20	37.82/5, n = 52
Postoperative MRC	469	− 0.769	0.442	33.95/5, n = 20	37.48/5, n = 52
Preoperative SRS	592	1.307	0.191	40.10/5, n = 20	34.39/5, n = 51
Postoperative SRS	512	0.032	0.975	36.10/5, n = 20	35.96/5, n = 51
Preoperative VRS	369	− 2.178	**0.029**	28.95/2, n = 20	39.40/3, n = 52
Postoperative VRS	524	0.195	0.845	36.70/1, n = 20	35.73/1, n = 51

*p* < 0.05*.* Significant *p* values between the two groups are written in bold.

*MRC* medical research council Scale for Muscle Strength [14], *SRS* sensory rating scale [15], *VRS* verbal rating scale for measuring pain intensity [16]

### Location category distribution of NF2- and SWNT-related schwannomas and association to parameters

In NF2-associated PNS, the median values for “preoperative MCR” (*H* (3) = 24.487, *p* < 0.001), “postoperative MCR” (*H* (4) = 18.877, *p* = 0.001), “preoperative SRS” (*H* (4) = 24.343, *p* < 0.001), “postoperative SRS” (*H* (4) = 21.787, *p* < 0.001) and “preoperative VRS” (*H* (4) = 17.499, *p* = *0.002*) were statistically significantly different between the categories.

No significance was seen in median values for “postoperative VRS” scores (*p* = 0.051) in NF2 cases and for all categories in SWNT-related PNS (*p* > 0.05).

In SWNT-related tumors, a non-significant tendency was seen for worse preoperative motor and sensory function. Intraspinal lesions seem to cause more pain before and all extremity schwannomas have less pain after surgery than other localizations.

**Fig. 1:**
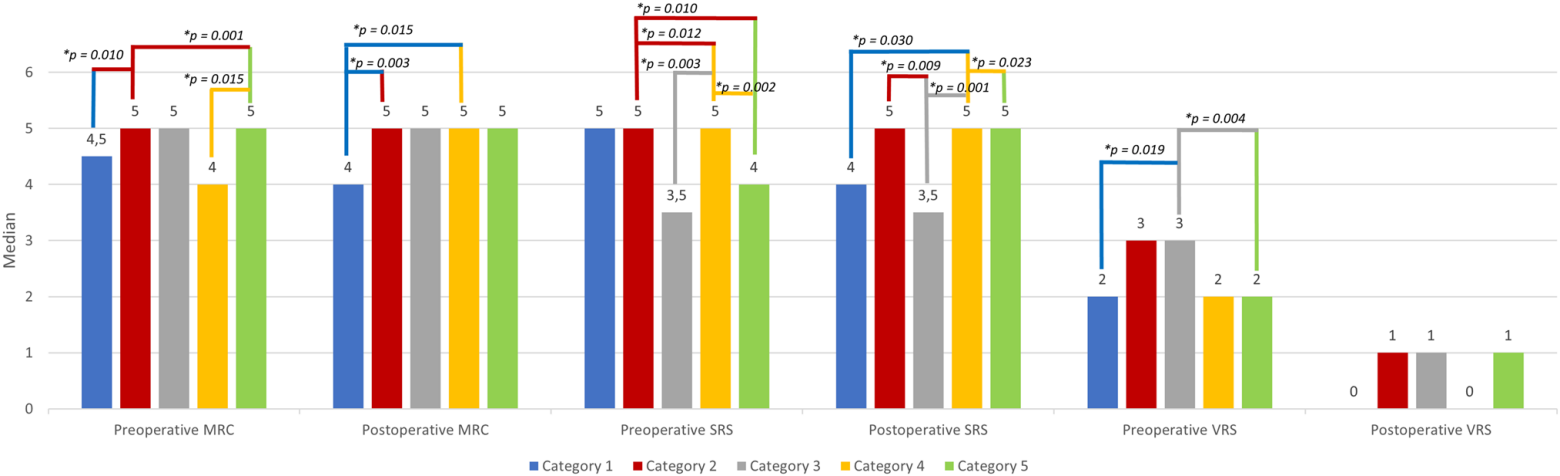
Significant group differences of pre- and postoperative motor and sensory function and pain intensity in NF2-associated peripheral and spinal nerve schwannomas. No significant group difference was observed in SWNT-associated tumors in any of the functional categories/pain category. *Note* Category 1: Head/face/neck/brachial plexus; Category 2: Upper extremity; Category 3: Lower extremity; Category 4: Torso (thorax/abdomen/pelvic/back); Category 5: intraspinal

Subsequently, pairwise comparisons were performed using Dunn’s (1964) procedure with a Bonferroni correction for multiple comparisons. This post hoc analysis revealed statistically significant group differences only in NF2- and but not in SWNT-related tumors. For the NF2-associated cases, group differences are illustrated and highlighted in Fig. 1 (* adjusted *p*-values).

All other comparisons showed no statistically significant difference in the mentioned categories. In NF2-associated schwannomas with the same median values, intraspinal tumors exhibited a higher and therefore better preoperative MRC score compared to upper extremity (*p* = 0.001) cases but lower and thus worse postoperative SRS scores compared to tumors located at the torso (*p* = 0.023). In turn, the latter showed a higher SRS score preoperatively than those schwannomas located in the upper extremity (*p* = 0.012).

### Comparison between NF2- and SWNT-related schwannomas and association to parameters

Distributions of the pre-and postoperative MRC, SRS, and VRS scores were similar in the groups “NF2-associated” and “SWNT-associated”, as assessed by visual inspection. Median scores for preoperative SRS and VRS as well as Age at time of surgery and Resection amount were statistically significantly (*p* < 0.05) different between “NF2-associated” and “SWNT-associated” schwannomas. NF2-associated tumors exhibited more cases with affected sensory category levels (0–4) before surgery and partial resection amounts compared to SWNT-related cases. Detailed values are outlined in Table 4.

**Table 4:** The difference in pre-and postoperative functional parameters, pain sensation, and age at the time of surgery between patients with NF2-and SWNT-associated schwannomas

Variable	*U*	Z	*P*	Mean rank/median NF2-associated	Mean rank/median SWNT-associated
Preoperative MRC	4106	− 1.720	0.085	97.08/5, n = 130	109.47/5, n = 72
Postoperative MRC	4287	− 1.089	0.276	103.77/5, n = 130	96.04/5, n = 72
Preoperative SRS	3951	− 2.205	**0.027**	96.71/5, n = 133	113.35/5, n = 71
Postoperative SRS	4266	− 1.211	0.226	98.82/5, n = 132	107.91/5, n = 71
Preoperative VRS	3954	− 2.253	**0.024**	96.73/2, n = 133	114.58/3, n = 72
Postoperative VRS	4650	− 0.097	0.923	102.27/1, n = 132	101.50/1, n = 71
Age at time of surgery in years	1338	− 8.514	** < 0.01**	25 ± 11 n = 133	46 ± 14, n = 72
Resection amount	5319	2.172	**0.030**	99/1, n = 133	110.38/1, n = 72

*p* < 0.05*.* Significant *p* values between the two groups are written in bold.

*MRC* medical research council scale for muscle strength [14], *SRS* sensory rating scale [15], *VRS* verbal rating scale for measuring pain intensity [16]

Of the 201 tumors analyzed in the SRS category, the postoperative SRS category was higher in 21 tumors and lower in 20 tumors, and 162 unchanged in preoperative scores.

Of the 203 tumors analyzed in the VRS category, the postoperative VRS category was higher in 2 tumors, lower in 164 tumors, and 37 tumors unchanged to preoperative scores. There was no statistically significant difference in preoperative MRC, postoperative MRC, and SRS as well as VRS scoring between the two groups.

## Discussion

Peripheral nerve schwannomas (PNS), including intraspinal manifestations, are typical lesions in patients with NF2 and schwannomatosis and due to their multiplicity and other comorbidities, their management is more complex compared to sporadic cases. Besides ophthalmological symptoms or manifestations, PNS are one of the major presenting symptoms in children with NF2 and are therefore important for establishing the diagnosis [8]. Although most lesions are asymptomatic/slightly symptomatic and stable or only slowly growing, in some NF2 patients they can cause multiple and narrowing neurologic deficits that make the surgical treatment necessary.

Dermatome-oriented and radiating pain was the most common indication for surgery in both NF2- (48%) and SWNT-related (63%) cases and focal-neurological deficits were more frequently presented in NF2-related cases compared to SWNT-related tumors.

In a study by Mehta et al. [18], investigating 12 patients with NF2 involving 28 resected tumors (19 schwannomas, 2 hybrid tumors, 7 with unavailable data), the major symptom before surgery was pain, followed by weakness. 91% of operated cases were stable or improved in their motor function and only one patient worsened.

Our data confirmed that intracapsular complete surgical resection is a safe and low-risk technique for symptomatic NF2- and SWNT-associated PNS.

In both syndromes, postoperative neurological function remained stable or improved in 91–99% of cases. Particularly pain intensity improved postoperatively in the vast majority of NF2- (75%) and SWNT-(92%) associated tumors.

The fact that total resection extents were more often achieved in SWNT-associated PNS could be attributed to the fact that NF2 cases had more preoperative neurologic deficits, and thus surgical treatment was more restrictive to preserve function and not provoke a worsening. However, it is also interesting to hypothesize whether these tumors are intraoperatively more vulnerable to surgical manipulation.

In addition, the exact influence of the detailed resection extent needs to be further investigated within MRI- and or ultrasound-based volumetric studies. For the current study, MRI datasets are insufficient because superficial lesions, in particular, were predominantly examined by ultrasound. Measurement by volumetry requires a prospective study protocol, which is not given for the current retrospective data set but our group is currently in the process of designing such a study.The association between the genetic status on the surgical outcome of resected peripheral nerve schwannomas has not yet been reported in the literature.

Genotype–phenotype correlations are well studied and confirmed in NF2, contrary to SWNT. However, the influence of genetics on the outcome of therapy has not yet been adequately elucidated. An association is suspected [10, 19], but case numbers are too small, as in the presented cohort, and mosaic cases, in particular, should be re-examined using next-generation sequencing. Postulated is a higher mutation rate than previously demonstrated. In particular, the genetic diagnosis of schwannomatosis is not yet fully developed. In addition, a clear distinguishment between mosaic NF2 and SWNT cases is not often possible.

In the category comparison of NF2-associated peripheral schwannomas, lesions located at the trunk exhibited good functional and pain outcomes compared to tumors at the head/brachial plexus and neck. This can be explained by the fact that schwannomas in the trunk region were usually located superficially either subcutaneously or intramuscularly, for example along the intercostal nerves, and that few motor deficits were to be expected here. In comparison, the risk of functional damage is higher with tumors in the brachial plexus or intraspinal lesions, which are often not delineated and more difficult to prepare and resect. Here, the indication must be made very carefully. High-resolution nerve ultrasound [20] and MRI diagnostics help to assess the surgical risk by evaluating the demarcation to surrounding structures and tumor configuration.

Indication for surgery was most often based on severe or progressive functional impairment, drug-resistant pain, and rapid or massive tumor progression. For these cases, surgery is the treatment of choice and due to the possible variety of localizations often has to be carried out by an interdisciplinary team. The use of electrophysiology and ultrasound as well as neurosurgeons' experience and surgical technique also improves the postoperative outcome and minimizes the perioperative risks. Intracapsular resection is the favorite and only responsible technique in a benign tumor entity to minimize postoperative deficits [17, 18]. The reported low rate of complication (1%) and/or worsening (9%) independent of disease type can also be supported by our results using the same technique and does enable nevertheless a high rate of total resection in NF2 and SWNT patients.

Limitations to this study arise from the retrospective character of the analysis. Furthermore, NF2 and SWNT are rare genetic conditions and while the presented data set is the largest published to date the statistical power of our study is limited.

In addition, as already evident from the patient to tumor ratio, patients with multiple schwannomas in both disease types were included. However, there were clear differences between the localizations (categories) in terms of functional scale and pain localization (punctual or radiating pain). Nevertheless, these are the same patients and overlaps cannot be completely excluded. Of course, this must be taken into account when interpreting the results.

## Conclusion

The management of peripheral nerve schwannomas associated with NF2 and SWNT is complex due to multiplicity and comorbidities, prompting for interdisciplinary care. Indication for surgery is dictated by neurological deficits, drug-resistant pain, or significant tumor progression. Utilizing principles of microsurgery and interfascicular preparation, resection as associated with a very low complication rate and excellent results concerning pain reduction. Preoperative neurological impairment and total resection rate was higher in SWNT-associated PNS.

## Supplementary Information

Below is the link to the electronic supplementary material.

Supplementary file1 (DOCX 28 KB)

## Data Availability

The datasets analyzed during the current study are available from the corresponding author on reasonable request.
