# Supplementary material for: Management of neurofibromatosis type 2 and schwannomatosis associated peripheral and intraspinal schwannomas: influence of surgery, genetics, and localization
Source: J Neurooncol. 2022 Jun 30;159(2):271–9. doi: 10.1007/s11060-022-04061-0 (PMC9424169; doi:10.1007/s11060-022-04061-0)
Supplement: Supplementary file 1 — Supplementary file1 (DOCX 28 KB) [file 11060_2022_4061_MOESM1_ESM.docx]

**Supplementary Material**

**Supplementary Table** 5**.** Immediate pre-and postoperative functional rating scores in 133 operated NF2-associated non-intracranial schwannomas.

| **MRC Scale[14]** | Postoperative Class (No) | | | | |  |  |
| --- | --- | --- | --- | --- | --- | --- | --- |
| Preoperative Grade (No) | 0 (2) | 1 (3) | 2 (4) | 3 (19) | 4 (15) | 5 (87) | NA (3) |
| 0: No visible contraction (1)  1: Visible contraction without movement of the limb (5)  2: Movement of the limb but not against gravity (10)  3: Movement against gravity over (almost) the full range (15)  4: Movement against gravity and resistance (17)  5: Normal (82)  NA: Not available (3) | 1  0  0  0  0  1  0 | 0  3  0  0  0  0  0 | 0  1  2  1  0  0  0 | 0  1  7  9  1  1  0 | 0  0  1  2  11  1  0 | 0  0  0  3  5  79  0 | 0  0  0  0  0  0  3 |
| **SRS Scale** | Postoperative Class (No) | | | | |  |  |
| Preoperative Grade (No) | 0 (0) | 1 (21) | 2 (7) | 3 (8) | 4 (20) | 5 (76) | NA (1) |
| 0: Anesthesia (1)  1: Severe Hypesthesia (28)  2: Severe Paresthesia (5)  3: Moderate Paresthesia (2)  4: Slight Paresthesia (20)  5: No sensory deficit (77) | 0  0  0  0  0  0 | 0  21  0  0  0  0 | 0  2  4  0  0  1 | 1  4  1  0  1  1 | 0  0  0  2  16  2 | 0  0  0  0  3  73 | 0  1  0  0  0  0 |
| **VRS (0-3)** | Postoperative Class (No) | | | | |  |  |
| Preoperative Class (No) | 0 (65) | 1 (43) | 2 (23) | 3 (1) | NA (1) |  |  |
| 0: No pain (28)  1: Slight pain (9)  2: Moderate pain (31)  3: Severe pain (65) | 28  7  14  16 | 0  1  14  28 | 0  0  3  20 | 0  1  0  0 | 0  0  0  1 |  |  |

**Note.** No – Number; MRC – Medical Research Council Scale for Muscle Strength [14]; SRS – Sensory Rating Scale [15]; VRS – Verbal Rating Scale for measuring pain intensity [16].

**Supplementary Table** 6**.** Immediate pre-and postoperative functional rating scores in 72 operated SWNT-associated non-intracranial schwannomas.

| **MRC Scale[14]** | Postoperative Class (No) | | | | |  |  | |
| --- | --- | --- | --- | --- | --- | --- | --- | --- |
| Preoperative Grade (No) | 0 (4) | 1 (1) | 2 (1) | 3 (1) | 4 (14) | 5 (50) | NA (1) | |
| 0 (1)  1 (1)  2 (2)  3 (2)  4 (14)  5 (52) | 1 0  0  0  0  3 | 0 0  1  0  0  0 | 0 0  0  0  0  1 | 0 0  0  0  1  0 | 0 1  1  1  11  0 | 0 0  0 1  2 47 | 0  0  0  0  0  1 | |
| **SRS Scale** | Postoperative Class (No) | | | | |  |  |  |
| Preoperative Grade (No) | 0 (0) | 1 (15) | 2 (0) | 3 (2) | 4 (4) | 5 (50) | NA (1) |  |
| 0 (0)  1 (15)  2 (3)  3 (3)  4 (3)  5 (48) | 0  0  0  0  0  0 | 0  14  1  0  0  0 | 0  0  0  0  0  0 | 0  0  0  2  0  0 | 0  1  1  1  0  1 | 0  0  1  0  3  46 | 0  0  0  0  0  1 |  |
| **VRS (0-3)** | Postoperative Class (No) | | | | |  |  |  |
| Preoperative Class (No) | 0 (34) | 1 (26) | 2 (10) | 3 (1) | NA (1) |  |  |  |
| 0 (4)  1 (1)  2 (25)  3 (42) | 3  1  17  13 | 1  0  7  18 | 0  0  1  9 | 0  0  0  1 | 0  0  0  1 |  |  |  |

**Note.** No – Number; MRC – Medical Research Council Scale for Muscle Strength [14]; SRS – Sensory Rating Scale [15]; VRS – Verbal Rating Scale for measuring pain intensity [16].
